# Fecal microbiota changes in people with cystic fibrosis after 6 months of elexacaftor/tezacaftor/ivacaftor: Findings from the promise study

**DOI:** 10.1016/j.jcf.2025.05.006

**Published:** 2025-06-06

**Authors:** Jennifer T Duong, Hillary S Hayden, Adrian J Verster, Christopher E Pope, Carson Miller, Kelsi Penewit, Stephen J Salipante, Steven M Rowe, George M Solomon, David Nichols, Andrea Kelly, Sarah Jane Schwarzenberg, Steven D Freedman, Lucas R Hoffman

**Affiliations:** a Department of Pediatrics, Division of Pediatric Gastroenterology, Hepatology, and Nutrition, University of California San Francisco School of Medicine, San Francisco, CA, USA; b Department of Microbiology, University of Washington School of Medicine, Seattle, WA, USA; c Pythia Informatics, Ottawa, Canada; d Department of Laboratory Medicine and Pathology, University of Washington School of Medicine, Seattle, WA, USA; e Department of Medicine and the Gregory Fleming James Cystic Fibrosis Research Center, University of Alabama at Birmingham, Birmingham, AL, USA and CF Foundation, Bethesda, MD, USA; f CF Foundation, Bethesda, MD, USA and University of Washington School of Medicine, Seattle, WA, USA; g Department of Pediatrics, University of Pennsylvania Perelman School of Medicine, Philadelphia, PA, USA; h Department of Pediatrics, University of Minnesota Masonic Children’s Hospital, Minneapolis, MN, USA; i Beth Israel Deaconess Medical Center, Harvard Medical School, Boston, MA, USA; j Department of Microbiology and Department of Pediatrics, Division of Pulmonary and Sleep Medicine, University of Washington School of Medicine, Seattle, WA, USA

**Keywords:** Dysbiosis, Fecal microbiome, Fecal calprotectin, Elexacaftor/tezacaftor/ivacaftor

## Abstract

**Background:**

People with cystic fibrosis (PwCF) often have fecal dysbioses relative to those without CF, characterized by increased pro-inflammatory microbiota and gastrointestinal (GI) inflammation as measured by fecal calprotectin, suggesting that inflammation contributes to CF GI disease. The multicenter observational PROMISE study (NCT04038047) found that calprotectin decreased in PwCF treated with elexacaftor/tezacaftor/ivacaftor (ETI). To better understand the dynamics between fecal dysbiosis and GI inflammation, we characterized the microbiomes of fecal samples from PROMISE and the relationships with calprotectin before, 1-month post, and 6-months post ETI.

**Methods:**

Fecal microbiota from participants ≥12 y/o were determined by shotgun metagenomic sequencing with random forest modeling and multivariate linear regression analysis to define relationships between microbiota, calprotectin, and deltaF508 genotype before and after ETI.

**Results:**

We analyzed 345 samples from 124 participants. At baseline, we observed community-level differences in the fecal microbiota among participants with abnormal compared to normal calprotectin. With ETI, the relative abundances of 7 bacterial species – *Escherichia coli, Staphylococcus aureus, Clostridium scindens, Enterocloster clostridioformis, Clostridium butyricum, Anaeroglobus geminatus*, and *Ruminococcus gnavus* – decreased significantly, correlating with calprotectin decrease. We detected community-level differences in the fecal microbiota based on CFTR genotype and a distinct pattern of microbiota change in F508del homozygous compared to heterozygous participants after ETI.

**Conclusions:**

We identified 7 species for which fecal abundances decreased with ETI and correlated with calprotectin decrease, supporting a close relationship between fecal microbiota and inflammation in PwCF. Future work will define these relationships with metabolites and GI symptoms during long-term ETI therapy

## 1. Background

Cystic fibrosis (CF) is an autosomal recessive genetic disorder caused by mutations in the CF transmembrane regulator (CFTR) protein, which leads to altered ion and fluid transport across epithelial membranes in multiple organ systems [1]. Gastrointestinal (GI) complications are a significant cause of morbidity and mortality for people with CF (PwCF), including intestinal obstruction, pancreatic insufficiency, and CF-associated liver disease [2–4]. While the pathogenesis of these GI manifestations is incompletely understood, there is a hypothesized role for intestinal inflammation as a driver of GI disease. Multiple human studies in PwCF have demonstrated intestinal inflammation, often identified by increased fecal calprotectin [5,6], in association with GI disease manifestations, including endoscopically observed intestinal lesions [6], GI malignancy [7], poor growth [5], and CF-associated liver disease [8].

Understanding the causes and consequences of intestinal inflammation in the CF GI tract is therefore critical to elucidating the pathogenesis of CF-related GI disease. Multiple studies have identified differences in the fecal microbiome, often referred to as dysbioses, in PwCF compared to healthy individuals without CF, both in terms of taxonomic composition (microbiota) and bacterial functional capacities (metagenomes) [9–11]. It is hypothesized that these CF dysbioses are due to abnormalities in the CF GI tract resulting from CFTR dysfunction, including thickened mucus, fat malabsorption, low intestinal pH, and intestinal dysmotility, in combination with high fat diets and frequent antibiotic exposure [3,4,12]. Several human studies have linked these CF fecal dysbioses with elevated fecal calprotectin [13,14] and other measures of GI dysfunction [8,14,15]. As compared to healthy individuals, PwCF have increased fecal abundances of pro-inflammatory microbiota, including species from the family Enterobacteriaceae and from the genera *Streptococcus, Veillonella*, and *Staphylococcus* [9–13,16].

In particular, multiple studies comparing PwCF to healthy individuals found higher fecal relative abundances of *Escherichia coli* that correlated directly with fecal calprotectin and stool fat content [14,15,17]. Evidence suggests that *E. coli* is particularly capable of colonizing the CF intestine, which has higher luminal fat and mucus compared to the healthy intestine. For instance, *E. coli* isolated from fecal samples from children with CF more efficiently metabolized glycerol, which is relatively abundant in the CF GI tract due to fat malabsorption, compared with isolates from control children [18]. Observations from mouse models indicated not only that *Escherichia* species are enriched in CF mouse microbiota [19], but that *E. coli* is particularly adept at colonizing inflamed GI mucosal surfaces, resulting in further induction of inflammation [20]. Despite this body of evidence linking *E. coli* dysbiosis to the CF GI tract, it remains unclear whether *E. coli* is a cause, or simply an indicator, of CF GI inflammation and disease.

The advent of CFTR modulators provides a unique opportunity to explore the effects of corrected CFTR on fecal calprotectin, fecal microbial compositions, and CF GI manifestations. Previous work demonstrated concurrent decreases in fecal measures of inflammation (calprotectin and M2-pyruvate kinase) and in abundances of Enterobacteriaceae upon treatment with the CFTR potentiator ivacaftor [21]. Elexacaftor/tezacaftor/ivacaftor (ETI) is a highly effective triple-combination CFTR modulator therapy and is currently approved for PwCF 2 years of age and older with at least one F508del mutation in the *CFTR* gene. In PwCF aged 12 years and older, fecal calprotectin was shown to significantly decrease following ETI treatment in two observational studies, PROMISE and RECOVER [22,23]. However, how changes in the fecal microbiota relate to GI inflammatory measures following ETI therapy is currently unknown.

The PROMISE study is an ongoing prospective, multi-center observational study investigating the effect of ETI on diverse disease manifestations in PwCF at least 12 years old with the F508del mutation. We defined the fecal microbiota and measured calprotectin among PROMISE participants before and after 6 months of ETI to help define the dynamic relationships between dysbiosis and inflammation in the CF GI tract. We hypothesized that abundances of pro-inflammatory microbiota, including *E. coli*, would differ in CF fecal samples from participants with high versus low levels of fecal inflammation, and that restoration of CFTR function with ETI would reduce fecal abundances of pro-inflammatory pathogens in parallel with fecal calprotectin relative to pre-treatment.

## 2. Methods

This was an ancillary analysis of fecal samples from participants in PROMISE (NCT04038047), a multi-center prospective observational study examining the clinical impact of ETI in PwCF ages ≥12 y/o with at least one F508del allele in the *CFTR* gene. Fifty-six US CF Foundation-accredited Care Centers in the CF Foundation Therapeutic Development Network participated, of which 18 centers participated in the GI sub-study that involved the collection of fecal samples. The study was approved by each center’s local Institutional Review Board. Full study details have been previously described [22,24,25].

Participants had a diagnosis of CF with *CFTR* mutations consistent with the FDA-labeled indication for elexacaftor/tezacaftor/ivacaftor (ETI) and had been prescribed or qualified for ETI. Full details regarding study participants, sample processing, measurement of fecal calprotectin, DNA extraction, analysis of the microbiota, and statistical analyses are provided in Supplemental Methods.

## 3. Results

### 3.1. Baseline (pre-ETI) demographics and clinical characteristics

We analyzed 345 samples from 124 PROMISE participants. Demographic and clinical characteristics of the study group are shown in Table 1. Of note, the median age of participants was 22.3 y/o, and most were heterozygous for the F508del mutation, pancreatic insufficient, and had previously been on modulators.

### 3.2. Changes during the study in the use of ursodeoxycholic acid and antibiotics

We first determined whether the use of common CF treatments recorded by PROMISE that were most likely to confound the current study results varied significantly over the three visits (pre-ETI and 1 and 6 months post ETI) (Supplemental Table 1). We identified no significant differences among visits in usage of ursodeoxycholic acid, inhaled antibiotics, and oral antibiotics. Participants had statistically higher use of acute antibiotics at 1 month post ETI compared to pre-ETI and 6 months post ETI. Information regarding laxative and acid suppression use was collected at baseline, but not over the course of the study, and therefore was not analyzed.

### 3.3. Relationship between fecal microbiota and fecal calprotectin

**Fig. 1.**
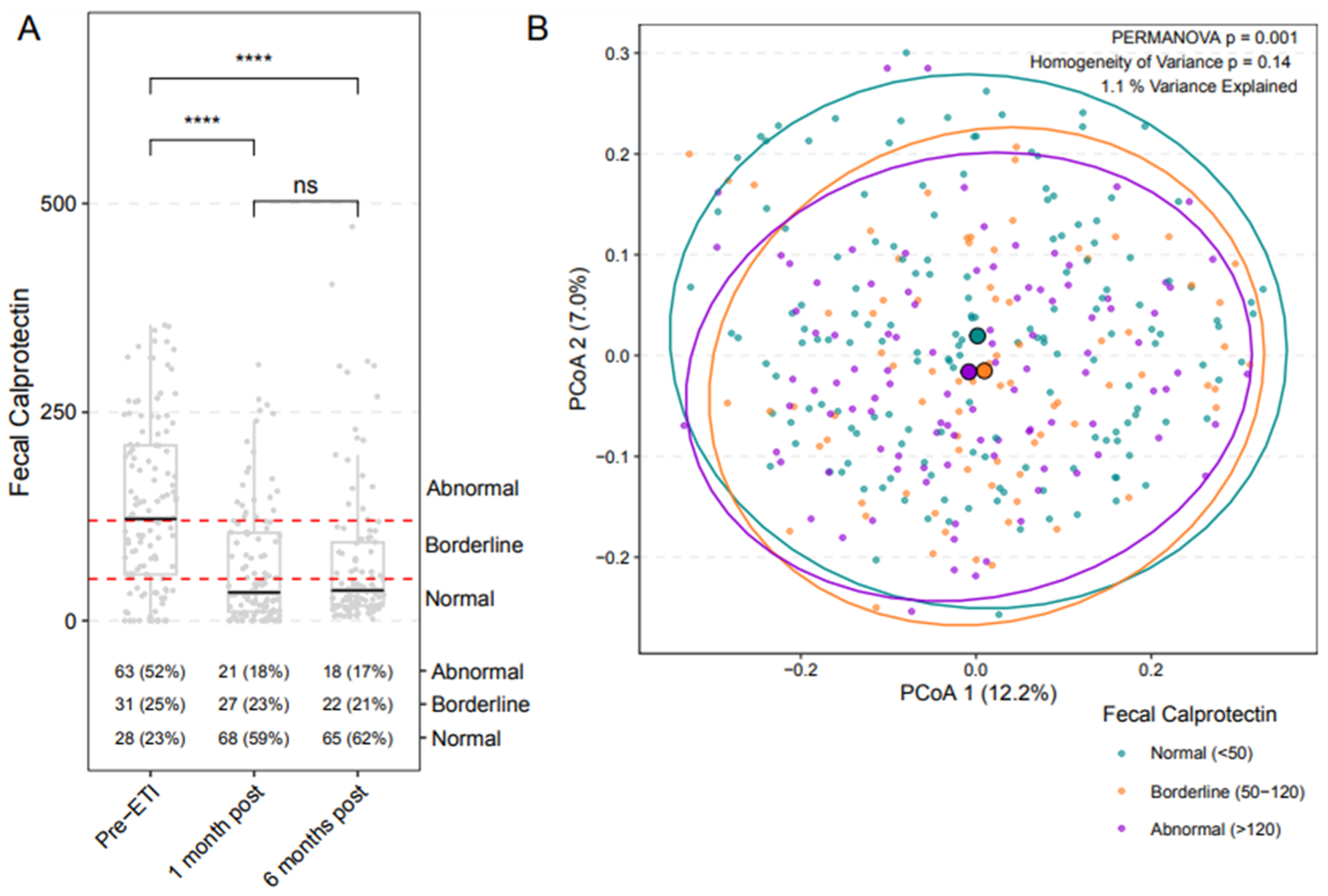
Differences in the fecal microbiota among participants with abnormal values of fecal calprotectin as compared to those with normal calprotectin. (A) Boxplot of fecal calprotectin among all participants pre-ETI and 1 and 6 months post ETI. Dotted red lines denote levels of fecal calprotectin with values <50 μg/g indicating normal; 50 – 120 μg/g, borderline; and >120 μg/g, abnormal. P-values were calculated from a quantile mixed effects model, specifically testing the median value in a log transformed space. (ns, not significant; ****p ≤ 0.0001) (B) Principal coordinates analysis (PCoA) utilizing Bray-Curtis dissimilarity demonstrating fecal microbiota of all samples categorized according to calprotectin (normal *n* = 161, borderline *n* = 80, and abnormal *n* = 102). Larger symbols represent centroids (average microbiota) for each category; smaller symbols represent individual sample microbiota. Ellipses indicate distinct clustering of normal, borderline, and abnormal fecal calprotectin samples. (PERMANOVA *p* = 0.001; homogeneity of variance *p* = 0.14). For results of ${\text{T}}_{\text{W}}^{2}$ test, see Supplemental Table 2.

Compared to baseline, fecal calprotectin was significantly lower at 1 and 6 months post ETI for the 124 subjects in this analysis (Fig. 1A). There was no significant change in fecal calprotectin comparing 1 month to 6 months post ETI. Greater detail regarding the change in fecal calprotectin following ETI initiation can be found in the PROMISE GI substudy report [22]. We defined the community-level characteristics of the fecal microbiota using principal coordinates analyses (PCoA) with Bray-Curtis dissimilarity as the distance metric (Fig. 1B). Analyzing samples from all participants from all three timepoints (pre-ETI, 1-month post, and 6-months post ETI), we detected significant differences in overall fecal microbiota composition between samples categorized by level of fecal calprotectin, as defined by values <50 μg/g, indicating normal; 50 – 120 μg/g, borderline; and >120 μg/g, abnormal (*p* = 0.001, PERMANOVA) (*p* = 0.14, homogeneity of variance). Specifically, we identified differences in the fecal microbiota among participants with abnormal values of fecal calprotectin compared to those with normal calprotectin (see Supplemental Table 2 for results of ${\text{T}}_{\text{W}}^{2}$ test). Vector arrows indicating the taxa contributing most to differences in the microbiota are shown in Supplemental Fig. 1.

### 3.4. Relationship between specific taxon abundances and fecal calprotectin

**Fig. 2.**
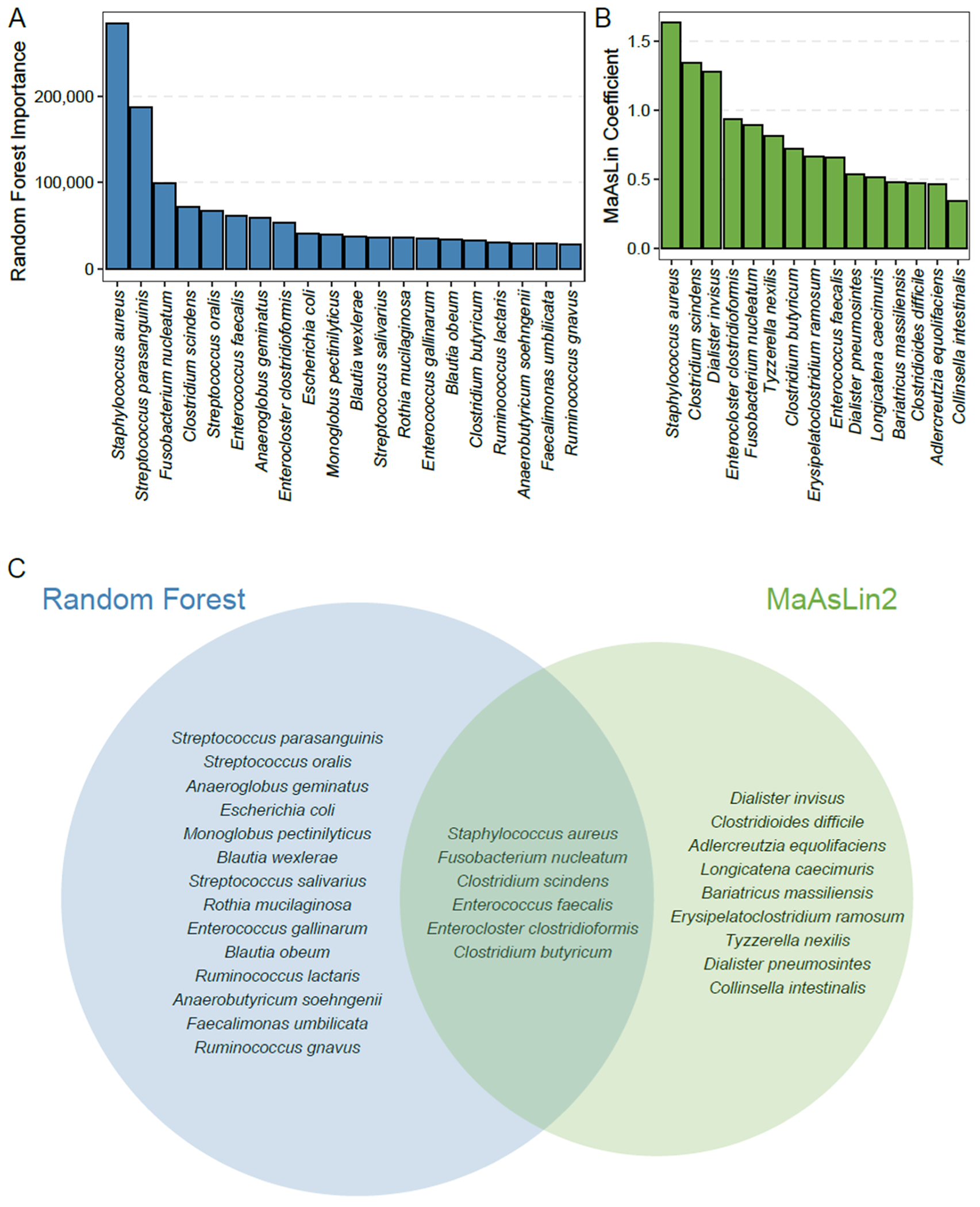
Taxa significantly associated with fecal calprotectin by Random Forest and MaAsLin2. (A) Bar plot demonstrating taxa contributing significantly to random forest model performance in a combined point dataset. Importance scores are indicated on the y axis and taxa (shown at the species level) on the x axis. (B) Bar plot demonstrating taxa with false discovery rate (FDR)-corrected P value <0.05 contributing significantly to MaAsLin2 performance in a combined time point dataset. Coefficient values are indicated on the y axis and species on the x axis. (C) Venn diagram demonstrating species uniquely identified as contributing to MaAsLin2 and Random Forest model performance, and the overlapping species between the two models.

Previous work demonstrated associations between higher relative abundances of Enterobacteriaceae, particularly *E. coli*, with greater measures of fecal calprotectin [14,15]. In this study, random forest and MaAsLin2 models were used to identify species significantly associated with fecal calprotectin (Fig. 2A and B). As demonstrated in Supplemental Fig. 2, utilizing the random forest model, no single species could account for changes in fecal calprotectin, and constructing informative models required multiple species. Specifically, model performance increased significantly with the addition of more species, ultimately saturating with the addition of approximately 20 species. This was true for models generated by two separate computational approaches. For these models, fecal calprotectin was utilized as a continuous variable to determine the association between calprotectin and the fecal microbiota without considering the clinical significance of the calprotectin values. We also generated an alternative categorical model that discriminated normal and borderline samples from those with abnormal calprotectin (below and above 120 μg/g). This categorical model was able to discriminate these two groups in cross validation (area under the ROC curve 0.72) and revealed a similar set of species contributing to changes in calprotectin as our continuous model, with 17 of the top 20 species overlapping.

Bacterial species that contributed most to the models generated by these two approaches are shown in Fig. 2C. Six species were included in models from both approaches: *Staphylococcus aureus, Fusobacterium nucleatum, Clostridium scindens, Enterococcus faecalis, Enterocloster clostridioformis*, and *Clostridium butyricum*. Interestingly, *E. coli* was identified as a relatively minor contributor to only one of the two models (Random Forest), perhaps related to the relatively lower abundances of Proteobacteria such as *E. coli* in fecal samples from adults with CF compared with samples from children with CF investigated in earlier studies of calprotectin and fecal dysbiosis [13,14].

### 3.5. Differences in fecal microbiota are observed at 6 months of ETI compared to baseline and 1 month of ETI

**Fig. 3.**
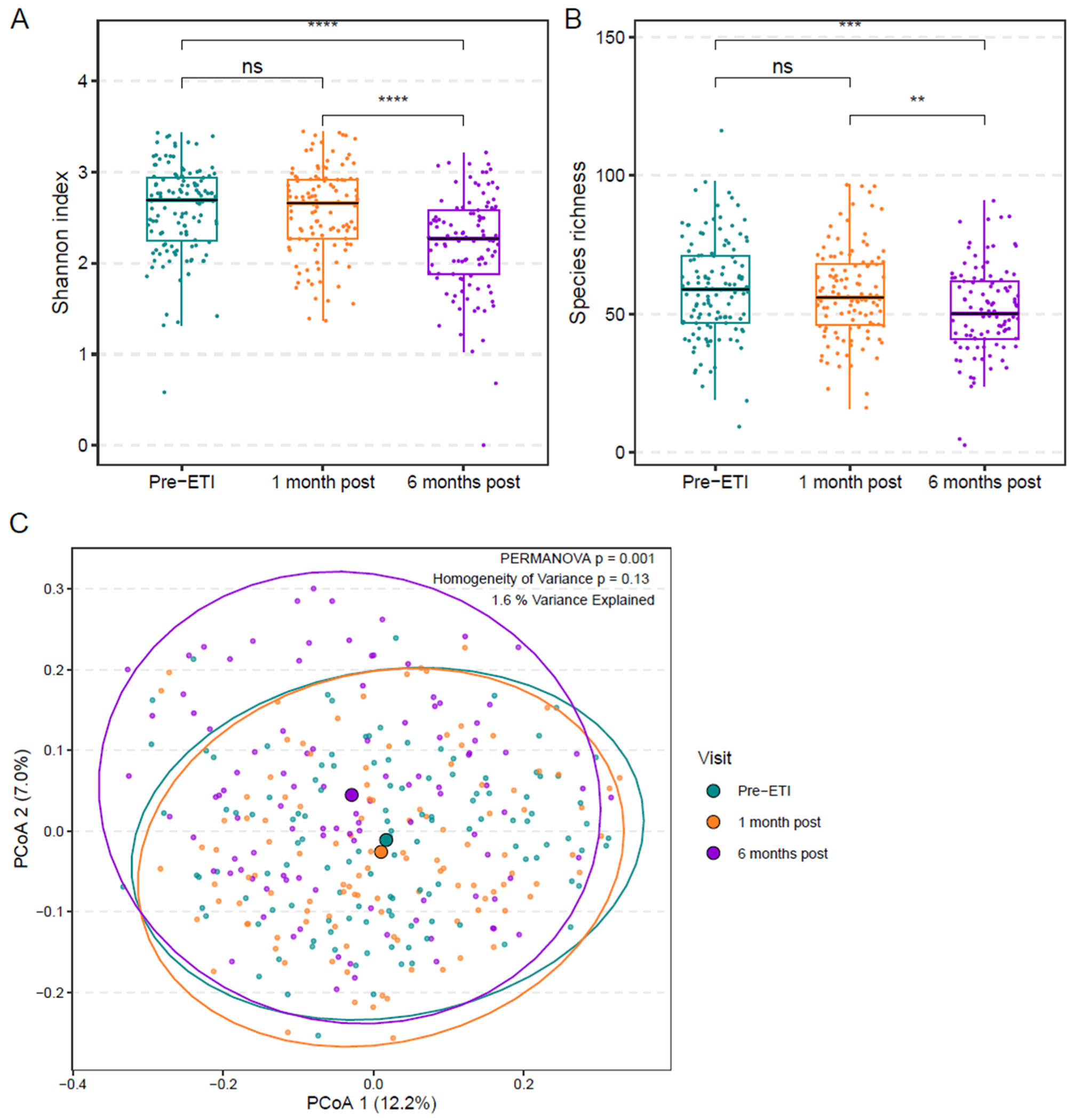
Differences in the fecal microbiota among participants at 6 months of ETI compared to baseline and 1 month of ETI. (A) Boxplot of Shannon Index with individual data points pre-ETI (*n* = 124), 1 month post (*n* = 116), and 6 months post (*n* = 105). The line indicates the median; boxplot hinges indicate the first and third quartiles. (ns, not significant; ****, *p* ≤ 0.0001; mixed effects model). (B) Boxplot of Species Richness with individual data points. (ns, not significant; **, *p* ≤ 0.01; ***, *p* ≤ 0.001). (C) Principal coordinates analysis (PCoA) utilizing Bray-Curtis dissimilarity demonstrating fecal microbiota pre-ETI and at 1 and 6 months post ETI. Larger symbols represent centroids (average microbiota); smaller symbols represent individual sample microbiota. Ellipses and results of ${\text{T}}_{\text{W}}^{2}$ test (see Supplemental Table 3) indicate distinct clustering of 6 months post ETI initiation from pre-ETI and 1 month post ETI initiation samples. (*p* = 0.001, PERMANOVA) (*p* = 0.13, homogeneity of variance).

Based on prior work by Ooi et al. investigating the fecal microbiota following ivacaftor [21] and Duong et al. following ETI [26], we hypothesized that within-sample microbial diversity (α-diversity) would not significantly change after ETI. To test this hypothesis, we calculated sample Shannon diversity indices and species richness. Participants were observed to have statistically lower fecal Shannon indices and species richness at 6 months post ETI compared to pre-ETI and 1-month post ETI (Fig. 3A and B). We compared community-level characteristics of the fecal microbiota on a PCoA using Bray-Curtis dissimilarity as in Fig. 1, this time comparing pre- and post ETI initiation timepoints (Fig. 3C). This analysis identified significant differences in overall fecal microbiota composition between samples at the three timepoints (pre-ETI, 1-month post, and 6-months post ETI) (*p* = 0.001, PERMANOVA) (*p* = 0.13, homogeneity of variance). Taxa contributing most to differences in the microbiota (Fig. 3C) are shown in Supplemental Fig. 3.

### 3.6. Treatment with ETI was associated with decreases in fecal abundances of seven species

**Fig. 4.**
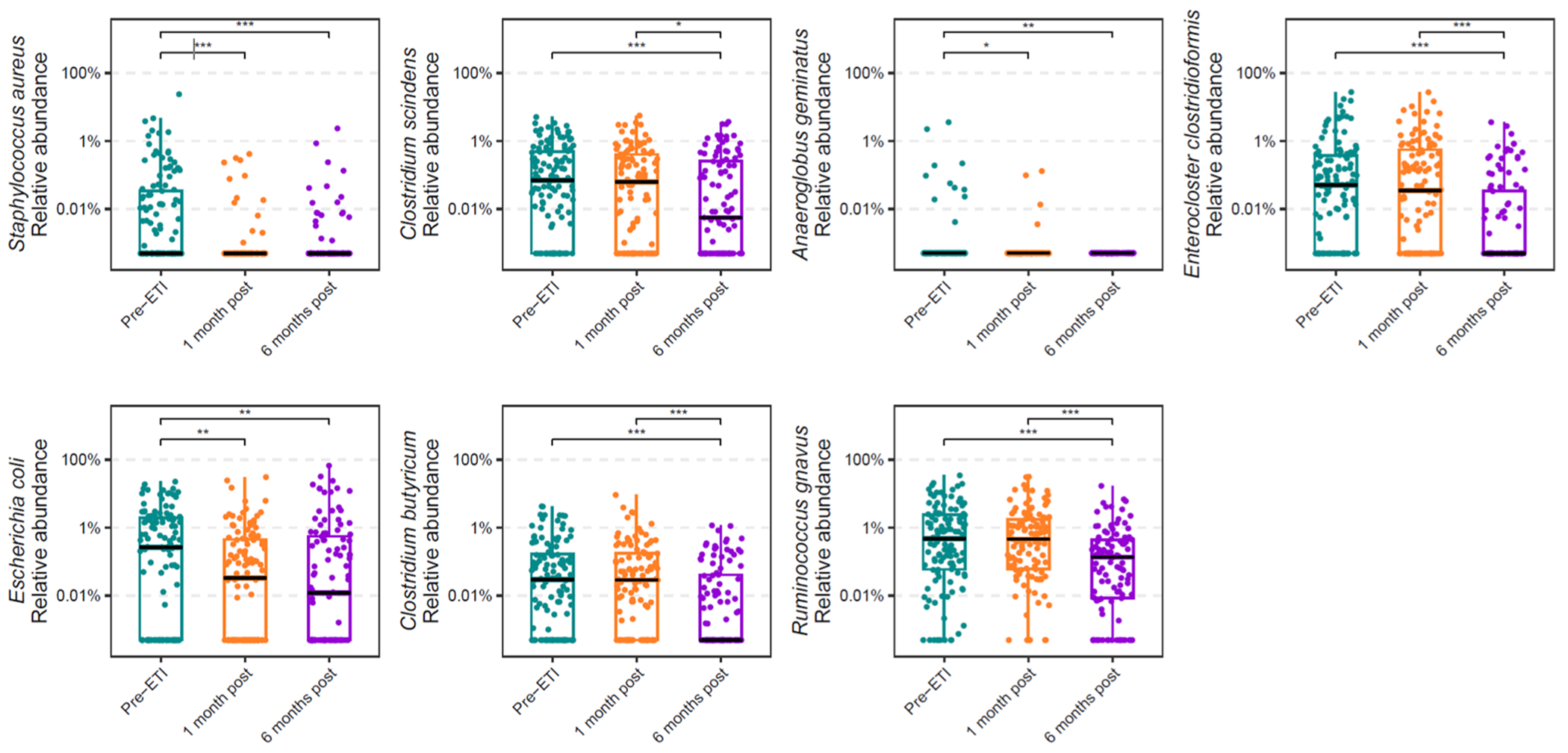
Relative abundances of 7 species decreased significantly after 6 months of ETI as compared to pre-ETI. Boxplots indicating the relative abundances of the seven species associated with fecal calprotectin with statistically significant decrease during treatment with ETI (pre-ETI *n* = 124, 1 month post *n* = 116, and 6 months post *n* = 105). The black line indicates the median and the boxplot hinges indicate the first and third quartiles. Statistical testing was performed via quantile mixed effects models. Groups of microbes linked genomically (referred to as species-level genome bins, SGBs) but without species-level taxonomic characterization were excluded. Pairwise testing was performed via mixed effects model and demonstrated in Supplemental Table 4 (*, *p* ≤ 0.05; **, *p* ≤ 0.01; ***, *p* ≤ 0.001). For the relative abundances of the top twenty species associated with fecal calprotectin (identified based on random forest importance score) before and after treatment with ETI, see Supplemental Fig. 4.

Three hundred and forty-five samples were analyzed pre-ETI (*n* = 124), 1-month post ETI (*n* = 116), and 6 months post ETI (*n* = 105). Fig. 4 shows the relative abundances of the species associated with calprotectin with statistically significant difference between pre-ETI and at least one post ETI initiation timepoint. For the relative abundances of the top twenty species associated with fecal calprotectin (identified based on random forest importance score) before and after treatment with ETI, see Supplemental Fig. 4. We observed a significant decrease in relative abundances of 7 species – *Staphylococcus aureus, Clostridium scindens, Anaeroglobus geminatus, Enterocloster clostridioformis*, *E. coli*, *Clostridium butyricum, and Ruminococcus gnavus* – at least one timepoint post ETI initiation as compared to pre-ETI. Pairwise testing was performed via mixed effects modeling and demonstrated in Supplemental Table 4.

Furthermore, at 6 months post ETI compared to pre-ETI, the proportion of samples in which *Staphylococcus aureus, Enterococcus faecalis, Anaeroglobus geminatus, Enterocloster clostridioformis, and Enterococcus gallinarum* were detected declined, while the proportion of samples in which *Blautia wexlerae* was detected increased (Supplemental Fig. 5). Notably, samples with detectable *S. aureus* and *E. clostridioformis* decreased by 50 % and *A. geminatus* by 100 % with 6 months of ETI therapy.

### 3.7. Fecal relative abundances significantly decreased for five species and increased for one species after 6 months of ETI compared to pre-ETI in participants with calprotectin that was abnormal pre-ETI but normalized post ETI

Thirty eight of the 124 PROMISE participants (30.6 %) had an initially abnormal calprotectin pre-ETI that normalized by 1 or 6 months post ETI. Supplemental Fig. 6 shows the relative abundances of the twenty species most strongly associated with fecal calprotectin as identified by random forest modeling before and after treatment with ETI (Supplemental Fig. 6). Comparing 6 months of ETI relative to pre-ETI, we observed a significant decrease in fecal relative abundances of five species – *Staphylococcus aureus, Clostridium scindens, Anaeroglobus geminatus, Enterocloster clostridioformis*, and *Escherichia coli* – and a significant increase in relative abundances of *Blautia wexlerae* in participants with initially abnormal calprotectin that subsequently normalized. Pairwise testing was performed via mixed effects model and demonstrated in Supplemental Table 5.

### 3.8. Differences in fecal microbiota among participants with the F508del homozygous CFTR genotype compared to heterozygous G551D and minimal function genotypes

**Fig. 5.**
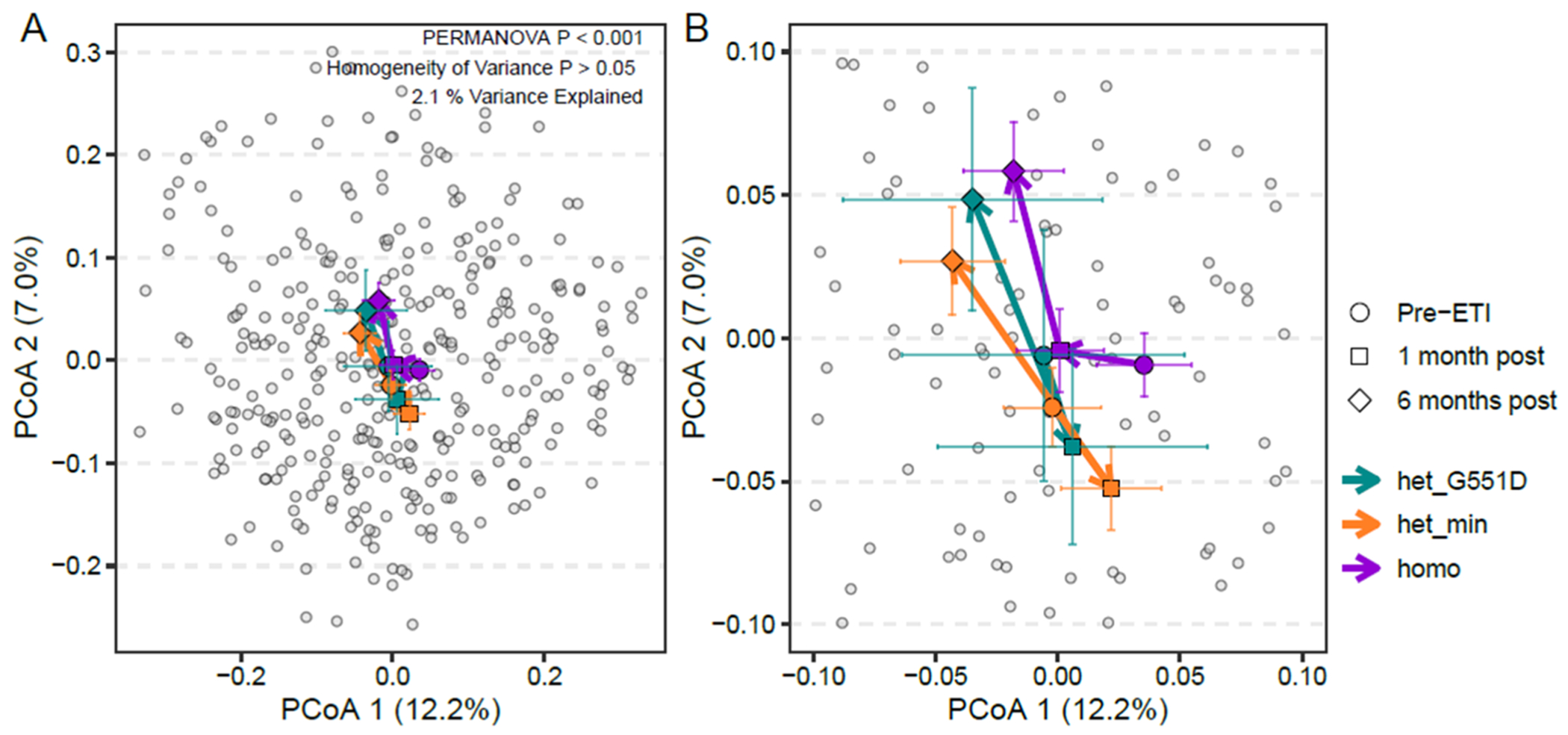
Differences in the fecal microbiota among participants with the F508del homozygous genotype compared to heterozygous genotypes. (A) PCoA utilizing Bray-Curtis dissimilarity demonstrating fecal microbiota of participants according to genotype pre-ETI (*n* = 124), and 1 (*n* = 116) and 6 months post ETI (*n* = 105). The colors indicate the three included genotypes. Homo, F508del homozygous. Het_G551D, heterozygous F508del, second mutation G551D. Het_min, heterozygous F508del, second mutation in the Vertex minimal function list for ETI eligibility [27]. Small symbols represent an individual sample’s microbiota. Larger symbols represent centroids (average microbiota) as classified by genotype at each of the three timepoints (pre-ETI and 1 and 6 months post ETI). The colored lines indicate the standard error, differences between genotype are statistically significant (*p* < 0.001, PERMANOVA) (*p* > 0.05, homogeneity of variance). (B) A larger graphical representation of the PCoA in Panel A.

As in our above analyses, we used PCoA using Bray-Curtis dissimilarity as the distance metric to compare fecal microbiota, this time among subjects categorized by CFTR genotypes (Fig. 5A and in a larger graphical representation in Fig. 5B). Analyzing samples from all participants from all three timepoints, we identified significant differences in overall fecal microbiota composition between samples from participants categorized as F508del homozygous (participants with two F508del *CFTR* mutations), heterozygous G551D (one F508del mutation, the second G551D), and heterozygous minimal function (one F508del mutation, the second mutation in the Vertex minimal function list for ETI eligibility) [27]. Fecal microbiota among F508del homozygous participants were different compared to heterozygous G551D and heterozygous minimal function participants, particularly at 1 month post ETI.

## 4. Discussion

Using samples from a multi-center, observational study of adults with CF, we identified 7 bacterial species for which fecal relative abundances decreased significantly with ETI, a highly effective CFTR modulator, and that directly correlated with a fecal measure of inflammation, calprotectin, supporting a strong pathophysiologic relationship between CFTR function, intestinal inflammation, and microbiota. While this observational study could not determine causal relationships among these three features of CF intestinal dysfunction, these findings identify bacterial taxa and activities that could contribute to CF GI symptoms, which have not responded as robustly to CFTR modulators as initially hoped [22].

Several studies of children with CF [14,15] and young adults [17] identified significant relationships between fecal calprotectin and alterations in the fecal microbiota, particularly increased abundances of *Escherichia coli* [14,15,17]. Recently, the PROMISE and RECOVER studies found participants to have significantly lower average fecal calprotectin with 6 and 12 months of ETI compared with pre-treatment values [22,23]. We therefore hypothesized that the fecal abundances of pro-inflammatory microbiota, including *E. coli*, would be increased in samples with high versus low level of fecal calprotectin, and that treatment with ETI would be followed by a reduction in both pro-inflammatory microbiota and, correspondingly, fecal calprotectin. We identified 7 bacterial species –*Escherichia coli, Staphylococcus aureus, Clostridium scindens, Enterocloster clostridioformis, Clostridium butyricum, Ruminococcus gnavus*, and *Anaeroglobus geminatus* — with decreased fecal abundances upon ETI treatment and that also correlated with decreased fecal calprotectin.

Of these, 5 species—*E. coli*, *S. aureus, E. clostridioformis, R. gnavus*, and *A. geminatus*—have been linked to CF as well as other GI conditions that involve GI inflammation, including inflammatory bowel disease (IBD), colorectal carcinoma (CRC), and liver disease. *S. aureus* is the most common traditional respiratory pathogen isolated from CF respiratory samples in the US [28] and was the most common pathogen cultured from sputum samples collected in PROMISE [29]. Furthermore, *S. aureus* was shown to have increased abundance in CF stool samples compared to healthy controls [30,31]. Of the 124 participants analyzed, 86 were found to have respiratory cultures positive for *S. aureus* based on Cystic Fibrosis Foundation Registry data. Of those 86, fifty-nine had *S. aureus* detected in the stool metagenomes. Earlier work has demonstrated a decrease in mean sputum densities of *S. aureus* following initiation of ETI [29,32]. Given the interconnectedness of the gut and lung in people with CF, future work is needed to characterize the airway microbiome during ETI treatment to determine if changes in *S. aureus* relative abundances in the stool mirror those in respiratory samples.

The abundances of *E. clostridioformis* [33] and *R. gnavus* [34–36] have been found to be higher in fecal and intestinal biopsy tissue from patients with IBD compared with healthy controls, corresponding to increased disease activity [34]. The abundance of *R. gnavus* [37] was also observed to be increased in intestinal biopsies associated with CRC compared with healthy tissue; accordingly, this taxon is thought to contribute to CRC pathogenesis through a series of inflammatory responses and oncogenic genetic modifications [36]. *A. geminatus*, an oral pathogen, was found to have increased fecal abundances in people with primary biliary cirrhosis compared to healthy individuals [38]. Therefore, as candidate pathogens in other clinical settings, the identification of these taxa in association with calprotectin was consistent with our hypothesis.

The remaining 2 species—*C. scindens* and *C. butyricum*—are generally thought to be commensal bacteria, with no known pathogenic potential and, in most cases, with activities that contribute to GI homeostasis, features that contrast with our hypothesis. *C. scindens*, a secondary bile acid-producing bacterium, has been linked to resistance to *C. difficile* infection due to its unique role in bile acid 7α-dehydroxylation [39]. *C. butyricum* can independently produce two short chain fatty acids (SCFAs), butyrate and acetate, which promote myriad features of GI health including intestinal barrier integrity, intestinal immune homeostasis, and protection against inflammation [40]. SCFAs have exhibited therapeutic promise when supplemented in murine models and human clinical trials of colitis, antibiotic associated diarrhea, and irritable bowel syndrome [41]. Therefore, *C. scindens* and *C. butyricum* are producers of key anti-inflammatory bacterial metabolites – secondary bile acids and SCFAs– raising questions about the roles of these taxa (if any) in CF GI disease and response to ETI. Future studies could investigate the relationships between these metabolites, and other features of the fecal metabolome, and their dynamic relationships with the fecal microbiota and calprotectin, following ETI.

Declines in the percentage of samples with detectable levels of these 7 bacterial species were striking. Detection of five species – *S. aureus*, *A. geminatus*, *E. clostridioformis, Enterococcus faecalis*, and *Enterococcus gallinarum*– declined significantly after 6 months of ETI compared to pretreatment. In particular, the proportion of samples with detectable *S. aureus* and *E. clostridioformis* decreased by 50 % and for *A. geminatus* by nearly 100 % with ETI. Rather than reflecting small shifts in the fecal microbiota, this pattern suggests a relatively marked decline in pro-inflammatory bacteria in PwCF following ETI. Additionally, this decline was more pronounced at 6 months compared to 1 month following ETI, indicating that changes in the CF GI tract may require longer treatment compared with other affected organs, such as the lung. This observation is consistent with previous work in PwCF and a *CFTR* gating mutation for whom initiating ivacaftor was followed by progressively improved fecal elastase, a measure of pancreatic sufficiency, with increasing duration of treatment [42,43]. Future work is required to determine if the microbial alterations observed here also persist or become more pronounced with longer duration of treatment, and how these alterations correlate with fecal calprotectin and other parameters of GI health, including fecal elastase, nutritional measures, and GI symptoms.

The PROMISE study primarily enrolled participants with three CFTR genotype categories, all with at least one F508del mutation – F508del homozygous, second mutation G551D, and a second minimal function mutation. Three participants (<3 % of the study total) had a fourth genotype – F508del heterozygous, but with the second mutation neither G551D nor minimal function; given the infrequency of this category, we focused our analysis on the three most common CFTR genotypes and did not include the fourth. Individuals with F508del homozygous and heterozygous G551D genotypes were generally eligible for previous CFTR modulators prior to the PROMISE study, while the majority of those with the heterozygous minimal function genotype were treatment naïve given the ineffectiveness of earlier modulator regimens (Supplemental Table 6). Given previous work showing varied impacts of earlier modulators on the fecal microbiome [21,44,45], we hypothesized that we would not detect significant differences in the fecal microbiota according to genotype categories. Ooi *et al.* identified significant changes in the fecal microbiota and a decrease in fecal calprotectin among individuals with G551D CFTR mutations with ivacaftor treatment [21]; however, other studies, including Ronan *et al.* examining ivacaftor in individuals with G551D mutations [44] and Pope et al. examining ivacaftor in individuals with R117H mutations and lumacaftor/ivacaftor in individuals with F508del mutations, did not detect significant changes in the fecal microbiome, intestinal inflammation, or pancreatic insufficiency following modulator treatment. Pre-ETI, we did not detect significant differences in overall fecal microbiota compositions between samples categorized by previous modulator use. We detected community-level differences in the fecal microbiota comparing participants categorized by genotype and a distinct pattern of fecal microbiota change in the F508del homozygous participants compared to the heterozygous G551D and minimal function genotypes. This finding suggests that ETI may impact fecal microbiota more than earlier-generation modulators do, and the effect of genotype and previous modulator use should be more carefully investigated in future work.

There are several limitations to this study. Treatment duration of 6 months was relatively short, and there may be further alterations in the fecal microbiota with increasing length of therapy. For instance, Marsh *et al.* observed an increase in core microbiota diversity and composition and a shift towards healthy controls with ETI therapy beyond 17 months [46]. Furthermore, our study did not control for diet or other medications. While we did not observe statistically significant differences in the use of ursodeoxycholic acid, inhaled antibiotics, and oral azithromycin at 1- and 6-months post ETI compared to pre-treatment, participants were observed to have statistically higher use of antibiotics for active infection at 1-month post ETI compared to the other timepoints. While statistically significant, the proportion of participants needing antibiotics remained clinically low, consistent with previous work showing a decrease in pulmonary exacerbations and antibiotic use following ETI [24,47]. Nonetheless, this treatment disparity could have impacted microbiota differences at 1-month post ETI compared to pre-treatment and 6-months post ETI. Furthermore, our study is limited by the sole use of relative abundance measurements, rather than a combination of relative and absolute abundance values, which could provide a more comprehensive assessment of total fecal microbial load and changes in the fecal microbiome following ETI initiation [48]. Finally, while this work strengthens the correlation between fecal dysbiosis and inflammation in CF following ETI, we are unable to establish causal relationships, which require laboratory studies to model the CF GI tract and relative dynamics of intestinal inflammation, the microbiota, and CFTR function.

In conclusion, these results demonstrate significant relationships between measures of intestinal inflammation and fecal microbiota, with concurrent changes in these features after 6 months of treatment with ETI. Additionally, our work identifies bacterial taxa that could contribute to CF GI symptoms, including bacterial species implicated in the generation of secondary bile acids and SCFAs. Future directions include further characterization of the fecal microbiota at later treatment timepoints, characterization of pre- and post ETI CF fecal metagenomes/functional capabilities and metabolomes with a focus on bile acids and SCFAs, and correlation of the above with markers of GI health, including fecal calprotectin, fecal elastase, nutritional measures, and GI symptoms.

## Supplementary Material

Supplemental file 1

Supplemental file 2

Supplementary material associated with this article can be found, in the online version, at doi:10.1016/j.jcf.2025.05.006.

## Figures and Tables

**Table 1:** Summary of baseline (pre-ETI) demographic and clinical characteristics of participants.

	Participants (*n* = 124)
Sex at Birth, Males - n (%)	66 (53.2)
Age (years) – Median (range)	22.3 (12.0–58.8)
Race, White – n (%)	118 (95.2)
F508del Genotype, second CFTR mutation, n (%)	
Homozygous	59 (47.6)
Heterozygous, G551D	11 (8.9)
Heterozygous, minimal function mutation	51 (41.1)
Heterozygous, not G551D or minimal function	3 (2.4)
Pancreatic Insufficiency – n (%)	120 (98.4)
Most Recent CFTR Modulator Use, n (%)	
None	57 (46)
Ivacaftor	11 (8.9)
Lumacaftor/ivacaftor	21 (16.9)
Tezacaftor/ivacaftor and ivacaftor	35 (28.2)
FEV_1_, % Predicted – Median (range)	89.2 (25.1–116.0)
BMI, kg/m^2^ – Median (range)	21.8 (15.2–29.8)
Use of Ursodeoxycholic Acid – n (%)	16 (12.9)
Use of Inhaled Antibiotics – n (%)	59 (47.6)
Use of Oral Azithromycin – n (%)	65 (52.4)
Acute Antibiotic Use – n (%)	0 (0)
Use of Osmotic or Stimulant Laxatives – n (%)	56 (45.2)
Use of Proton Pump Inhibitors – n (%)	45 (36.3)
Use of Histamine-2 Receptor Antagonists, n (%)	7 (5.6)
Fecal Calprotectin, μg/g stool – Median (range)	121.9 (0–354.3)
*S. aureus*-positive Respiratory Culture – n (%)	86 (69)

Abbreviations: F508del, delta F508; CFTR, cystic fibrosis transmembrane conductance regulator; FEV_1,_ forced expiratory volume in 1 s; BMI, body mass index; *S. aureus*, *Staphylococcus aureus*.

*S. aureus*-positive respiratory culture was defined by isolation of *S. aureus* from sputum or throat/nasal samples within 1 year prior to ETI initiation based on Cystic Fibrosis Foundation Registry data.

## Abbreviations:

BMI: body mass index
CF: cystic fibrosis
CFTR: cystic fibrosis transmembrane conductance regulator
ETI: elexacaftor/tezacaftor/ivacaftor
FDR: false discovery rate
FEV_1_: forced expiratory volume in 1 second
GI: gastrointestinal
PCoA: principal coordinates analysis
PwCF: people with cystic fibrosis
SCFA: short chain fatty acid
SGBs: species-level genome bins
ΔF508: F508del
