# Supplementary material for: Fecal microbiota changes in people with cystic fibrosis after 6 months of elexacaftor/tezacaftor/ivacaftor: Findings from the promise study
Source: J Cyst Fibros. Author manuscript; Available in PMC 2025 Aug 28. (PMC12393131; doi:10.1016/j.jcf.2025.05.006)
Supplement: Supplemental file 1 [file NIHMS2103854-supplement-Supplemental_file_1.docx]

**Supplemental Methods**

Study design and participants

Participants were clinically stable with no significant health changes within 14 days prior to their baseline visit, including no significant change in the use of medications such as acid suppression, antibiotics, pancreatic enzyme replacement therapy, probiotics, laxatives, and ursodeoxycholic acid. Exclusion criteria included participants with any significant health changes or the use of the following prior to the baseline visit: ETI within 180 days prior to baseline, acute antibiotics or systemic corticosteroids within 14 days, any new chronic therapy within 4 weeks, an investigational agent within 28 days, chronic oral corticosteroids within 28 days, or treatment of nontuberculous mycobacterial infection within 28 days. Additionally, history of lung or liver transplantation or organ transplantation listing precluded participants from the study.

Fecal sample collection

Fecal samples were collected from participants within 30 days prior (baseline), and 1 and 6 months after, initiating ETI. Participants collected samples at home using study kits, briefly stored them in freezers, then brought the samples to the clinics where they were stored frozen on site at -80°C. Samples were then shipped on dry ice to the University of Washington (Seattle, WA) for testing of fecal calprotectin, DNA extraction, and microbiome analysis. A total of 345 fecal samples were analyzed.

Measurement of fecal calprotectin

Measurement of fecal calprotectin was performed using 100 mg of fecal sample and the PhiCal ELISA Test (Genova Diagnostics, Inc, USA) according to the manufacturer’s instructions [1] [1,2], and as previously described for these samples [3].

DNA extraction

DNA extraction from fecal samples has been described previously [1] and was performed using the repeated bead beating plus column (RBB+C) methods as described [4] with the following modifications. To 250 grams of fecal sample, 1 mL of lysis buffer [500 mM NaCl, 50 mM Tris-HCl, pH 8.0, 50 mM EDTA, 4% sodium dodecyl sulfate (SDS)] and 0.5g of sterile, DNA/RNA-free silica/zirconia beads (Biospec) were added. Extraction was completed as previously described [4]. DNA was purified using the Qiagen DNeasy Blood & Tissue silica-based DNA extraction kit, then stored at -80°C until sequenced.

Shotgun metagenomic sequencing

For each sample, a random-fragment library was constructed using the Illumina DNA Prep Kit with dual indexing and sequenced on the NextSeq2000 platform using the NextSeq 1000/2000 P3 Reagents Sequencing Kit (Illumina). Sequencing generated an average of 26.0 million reads and 97.0% of samples had > 10 million reads after filtering. We quality-controlled our shotgun metagenomics data using fastp (v0.23.2) to remove reads shorter than 20 bases and with a quality lower than 12. We eliminated reads aligning to the human genome (assembly hg19) using bbduk.sh (v39.01) with parameters minid=0.95, maxindel=3, bwr=0.16, bw=12, quickmatch, fast, and minhits=2.

Taxonomic classification and relative species abundance of bacteria

Abundances of individual taxa in our metagenomic samples were quantified using MetaPhlAn (v.4.1.0, database mpa_vOct22_CHOCOPhlAnSGB_202212) [5] using default settings.

Statistical analysis

We excluded participants who contributed only a single fecal sample or without an initial visit, resulting in 124 participants with 345 samples for analysis. Summary statistics were used to describe demographic and clinical characteristics. For categorical variables, count and percent were calculated. For continuous variables, median and range were calculated.

Our data consists of multiple samples per participant, so to account for the non-independence of data points we used mixed-effects models where appropriate, with participant as a random effect. For calprotectin levels we applied a median mixed-effects model (using the lqmm function from the lqmm package with a quantile of 0.5) after log transformation. Alpha diversity was analyzed using a standard mixed-effects model (using the lmer function from the lme4 package). Bacterial abundance following ETI treatment was assessed using median mixed-effects models, while bacterial prevalence was evaluated with a binomial mixed-effects model (using the glmer function from the lmer package with a binomial family). Overall differences with respect to ETI treatment were tested with a post-ETI variable, while pairwise differences were calculated between each pair of visit using the emmeans function from the emmeans package. P-values for mixed-effects models were calculated using the lmerTest package. To control for multiple testing, we applied a false discovery rate (FDR) correction with a threshold of 0.05.

PCoA plots were created using the unweighted unifrac distance between samples, implemented by the phyloseq package in R. MetaPhlAn abundances of species-level genome bins (SGBs), along with a phylogenetic tree of SGBs (mpa_vOct22_CHOCOPhlAnSGB_202212.nwk), served as input to unifrac. Group separation significances were assessed using the PERMANOVA method (adonis2 function) and T_W_^2^ test (<https://github.com/alekseyenko/Tw2>) [6], and homogeneity of variances was tested using the betadisper function. Loading vectors were calculated with the biplot function in R. When analyzing beta diversity, there is no established method that accounts for multiple samples per individual, and such analyses violate the assumptions of the PERMANOVA test. With this limitation in mind, we anticipate that the calculated PERMANOVA p-values are lower than the actual. Those results should be interpreted alongside follow-up work with mixed effects models (from MaAsLin2 or otherwise), which do correct for multiple samples per individual.

Species associated with calprotectin were identified using either random forests (randomForest package) or MaAsLin2 (Maaslin2 package) [7]. We tested abundances of all taxonomically characterized species.

For random forest modeling, species abundance was log-transformed with a pseudo value of 10^-5^, and a model with 10,000 trees was trained. Species associated with calprotectin levels were identified by the top 20 importance scores. Model performance was assessed by the Spearman correlation between predicted and actual calprotectin levels in 10-fold cross-validation. Stepwise addition was used to determine the contributing species to the model by observing the saturation point of performance improvement.

MaAsLin2 employed built-in log transformation for species abundances. The model included calprotectin as the major fixed effect, with sex, age, ethnicity, genotype, prior modulator use, geographic site, and initial status as covariate fixed effects, and participant as a random effect. Associated bacteria were identified from calprotectin p-values, corrected for multiple tests using a false discovery rate of 0.05.

Data availability

All sequence read data generated for this research have been deposited in the Sequence Read Archive at the National Center for Biotechnology Information under BioProject ID: PRJNA1177516.

**References**

[1] Hoffman LR, Pope CE, Hayden HS, et al. Escherichia coli dysbiosis correlates with gastrointestinal dysfunction in children with cystic fibrosis. *Clin Infect Dis*. 2014;58(3):396-399. doi:10.1093/cid/cit715

[2] Lee JM, Leach ST, Katz T, Day AS, Jaffe A, Ooi CY. Update of faecal markers of inflammation in children with cystic fibrosis. *Mediators Inflamm*. 2012;2012:948367. doi:10.1155/2012/948367

[3] Borowitz D, Gelfond D. Intestinal complications of cystic fibrosis: *Current Opinion in Pulmonary Medicine*. 2013;19(6):676-680. doi:10.1097/MCP.0b013e3283659ef2

[4] Yu Z, Morrison M. Improved extraction of PCR-quality community DNA from digesta and fecal samples. *Biotechniques*. 2004;36(5):808-812. doi:10.2144/04365ST04

[5] Blanco-Míguez A, Beghini F, Cumbo F, et al. Extending and improving metagenomic taxonomic profiling with uncharacterized species using MetaPhlAn 4. *Nat Biotechnol*. 2023;41(11):1633-1644. doi:10.1038/s41587-023-01688-w

[6] Alekseyenko AV. Multivariate Welch *t* -test on distances. *Bioinformatics*. 2016;32(23):3552-3558. doi:10.1093/bioinformatics/btw524

[7] Mallick H, Rahnavard A, McIver LJ, et al. Multivariable association discovery in population-scale meta-omics studies. *PLoS Comput Biol*. 2021;17(11):e1009442. doi:10.1371/journal.pcbi.1009442
