# Supplementary material for: Fecal microbiota changes in people with cystic fibrosis after 6 months of elexacaftor/tezacaftor/ivacaftor: Findings from the promise study
Source: J Cyst Fibros. Author manuscript; Available in PMC 2025 Aug 28. (PMC12393131; doi:10.1016/j.jcf.2025.05.006)
Supplement: Supplemental file 2 [file NIHMS2103854-supplement-Supplemental_file_2.docx]

Supplemental Table 1. Summary of medication use over 6 months of ETI

|  | **Pre-ETI (n=124)** | **1 Month Post-ETI (n=116)** | **6 months Post-ETI (n=105)** | **P value** |
| --- | --- | --- | --- | --- |
| Ursodeoxycholic Acid | 16 (12.9) | 16 (13.8) | 12 (11.4) | >0.05 |
| Inhaled Antibiotics | 59 (47.6) | 57 (49.1) | 37 (35.2) | >0.05 |
| Oral Azithromycin | 65 (52.4) | 60 (51.7) | 51 (48.6) | >0.05 |
| Acute Antibiotic Use | 0 (0) | 7 (6.0) | 1 (1.0) | <0.05 |

Pearson's chi-squared test. Data shown as n (%)

Supplemental Table 2. Results of T_W_^2^ test comparing normal, borderline, and abnormal levels of fecal calprotectin

| **comparison** | **pval** | **p_adj** | **label** | **f_value** |
| --- | --- | --- | --- | --- |
| Normal - Borderline | 0.081 | 0.243 | P > 0.05 | 1.863839 |
| Normal - Abnormal | 0.002 | 0.006 | P < 0.01 | 1.863839 |
| Borderline - Abnormal | 0.334 | 1 | P > 0.05 | 1.863839 |

Supplemental Table 3. Results of T_W_^2^ test comparing pre-ETI, 1 month and 6 months post ETI

| **comparison** | **pval** | **p_adj** | **label** | **f_value** |
| --- | --- | --- | --- | --- |
| Pre-ETI - 1 month post | 0.029 | 0.087 | P > 0.05 | 2.815176 |
| Pre-ETI - 6 months post | 0.001 | 0.003 | P < 0.01 | 2.815176 |
| 1 month post - 6 months post | 0.001 | 0.003 | P < 0.01 | 2.815176 |

Supplemental Table 4. Results of pairwise post hoc tests comparing fecal relative abundances pre-ETI and 1 month and 6 months post ETI for all participants

| **species** | **contrast** | **P value** |
| --- | --- | --- |
| *Staphylococcus aureus* | (Pre-ETI) - 1 month post | <0.0001 |
|  | (Pre-ETI) - 6 months post | <0.0001 |
|  | 1 month post - 6 months post | >0.05 |
| *Clostridium scindens* | (Pre-ETI) - 1 month post | >0.05 |
|  | (Pre-ETI) - 6 months post | <0.001 |
|  | 1 month post - 6 months post | <0.05 |
| *Anaeroglobus geminatus* | (Pre-ETI) - 1 month post | <0.05 |
|  | (Pre-ETI) - 6 months post | <0.01 |
|  | 1 month post - 6 months post | >0.05 |
| *Enterocloster clostridioformis* | (Pre-ETI) - 1 month post | >0.05 |
|  | (Pre-ETI) - 6 months post | <0.0001 |
|  | 1 month post - 6 months post | <0.0001 |
| *Escherichia coli* | (Pre-ETI) - 1 month post | <0.01 |
|  | (Pre-ETI) - 6 months post | <0.01 |
|  | 1 month post - 6 months post | >0.05 |
| *Streptococcus salivarius* | (Pre-ETI) - 1 month post | >0.05 |
|  | (Pre-ETI) - 6 months post | >0.05 |
|  | 1 month post - 6 months post | >0.05 |
| *Clostridium butyricum* | (Pre-ETI) - 1 month post | >0.05 |
|  | (Pre-ETI) - 6 months post | <0.0001 |
|  | 1 month post - 6 months post | <0.0001 |
| *Ruminococcus gnavus* | (Pre-ETI) - 1 month post | >0.05 |
|  | (Pre-ETI) - 6 months post | <0.0001 |
|  | 1 month post - 6 months post | <0.0001 |

Supplemental Table 5. Results of pairwise post hoc tests comparing fecal relative abundances pre-ETI and 1 month and 6 months post ETI in participants with initially abnormal calprotectin which normalized

| **species** | **contrast** | **P value** |
| --- | --- | --- |
| *Staphylococcus aureus* | (Pre-ETI) - 1 month post | <0.001 |
|  | (Pre-ETI) - 6 months post | <0.01 |
|  | 1 month post - 6 months post | >0.05 |
| *Clostridium scindens* | (Pre-ETI) - 1 month post | >0.05 |
|  | (Pre-ETI) - 6 months post | <0.0001 |
|  | 1 month post - 6 months post | <0.05 |
| *Anaeroglobus geminatus* | (Pre-ETI) - 1 month post | <0.05 |
|  | (Pre-ETI) - 6 months post | <0.01 |
|  | 1 month post - 6 months post | >0.05 |
| *Enterocloster clostridioformis* | (Pre-ETI) - 1 month post | >0.05 |
|  | (Pre-ETI) - 6 months post | <0.0001 |
|  | 1 month post - 6 months post | <0.01 |
| *Escherichia coli* | (Pre-ETI) - 1 month post | >0.05 |
|  | (Pre-ETI) - 6 months post | >0.05 |
|  | 1 month post - 6 months post | >0.05 |
| *Blautia wexlerae* | (Pre-ETI) - 1 month post | >0.05 |
|  | (Pre-ETI) - 6 months post | >0.05 |
|  | 1 month post - 6 months post | >0.05 |

Supplemental Table 6. Previous Modulator Use by Genotype

| **ΔF508 Genotype** | **Previous Modulator Use – n (%)** |
| --- | --- |
| Homozygous (n=59) | Tezacaftor/ivacaftor and ivacaftor – 35 (59.3) |
|  | Lumacaftor/ivacaftor – 20 (33.9) |
|  | None – 4 (6.8) |
| Heterozygous G551D (n=11) | Ivacaftor – 11 (100) |
| Heterozygous minimal function (n=51) | Lumacaftor/ivacaftor – 1 (1.9) |
|  | None – 50 (98.1) |
| Heterozygous not G551D or minimal function (n=3) | None – 3 (100) |

Abbreviations: ΔF508, delta F508

|  |  |  |
| --- | --- | --- |


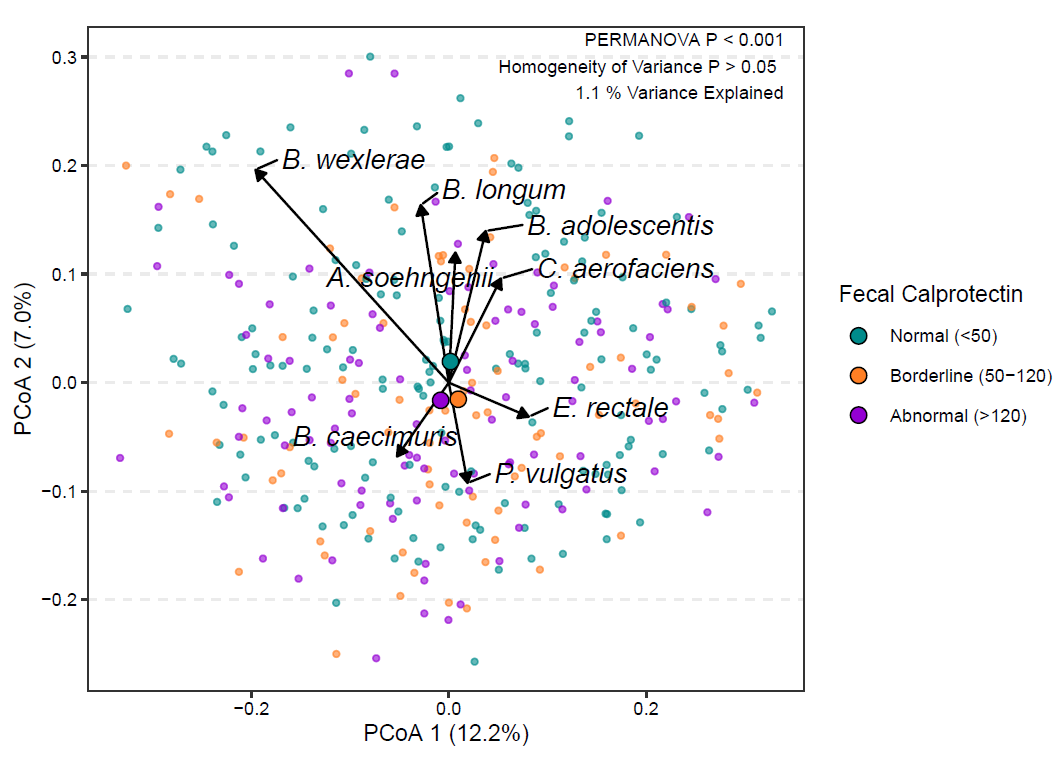


Supplemental Figure 1. Differences in the fecal microbiota are observed among participants with abnormal values of fecal calprotectin as compared to those with normal calprotectin

Principal coordinates analysis (PCoA) using Bray-Curtis dissimilarity demonstrating fecal microbiota of all participants from all timepoints with normal (n=161), borderline (n=80), and abnormal calprotectin (n=102) as in Figure 1B. Vector arrows (black) indicate taxa contributing most to differences in microbiota between all samples. Length of the vector denotes the relative contribution of the indicated taxon to inter-sample dissimilarities. The taxa identified include *Blautia wexlerae (B. wexlerae), Bifidobacterium longum (B. longum), Bifidobacterium adolescentis (B. adolescentis), Bacteroides caecimuris (B. caecimuris), Collinsella aerofaciens (C. aerofaciens), Eubacterium rectale (E. rectale), Phocaeicola vulgatus (P. vulgatus),* and *Anaerobutyricum soehngenii (A. soehngenii)*.


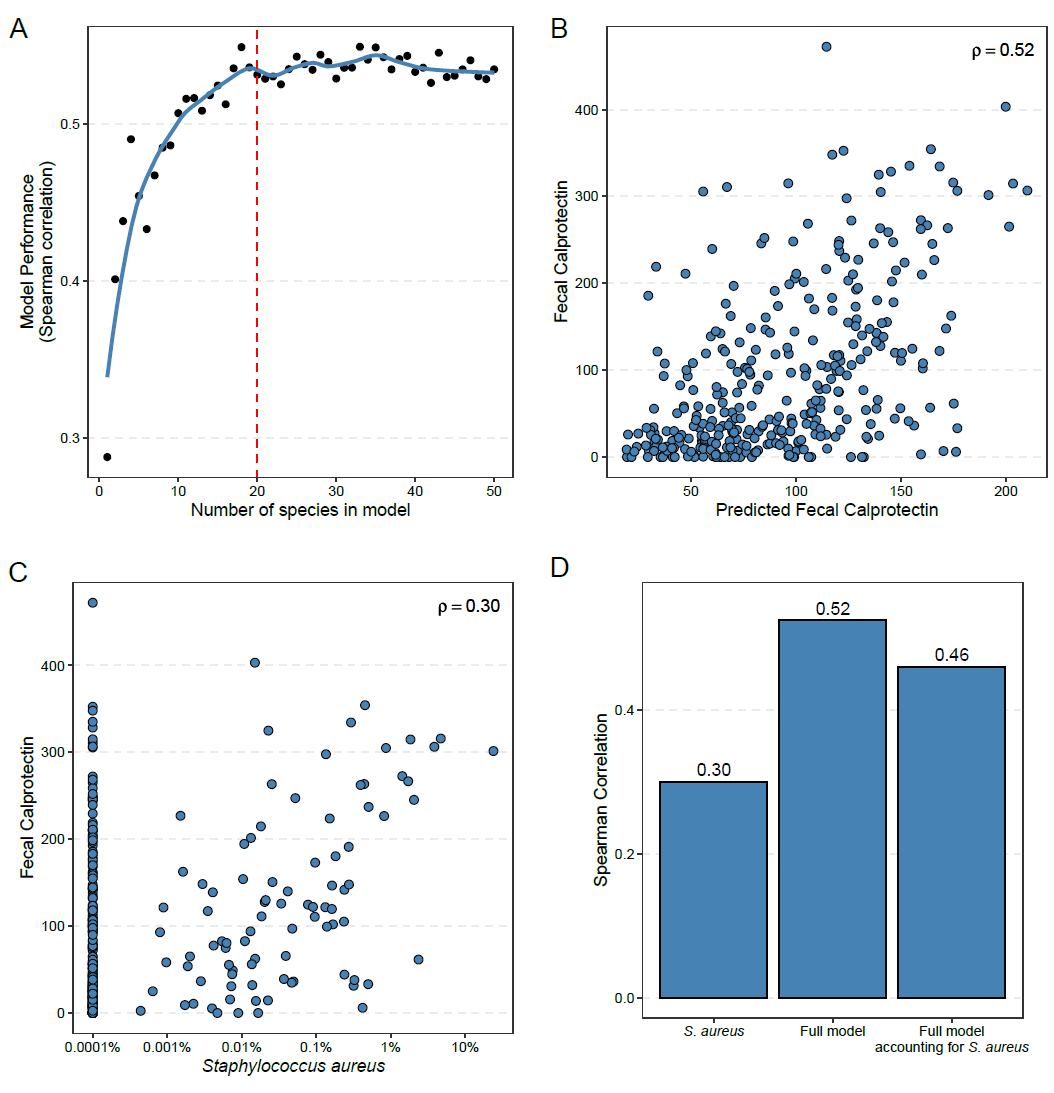


Supplemental Figure 2. Multiple species are required to account for changes in the abundance of fecal calprotectin utilizing the random forest model

(A) Scatter plot demonstrating the relationship between the performance of the random forest model in predicting the value of fecal calprotectin (as indicated by Spearman correlation on the y-axis) and number of species in the model (as indicated on the x-axis). As the number of species in the model increases, the model performance increases sharply, then saturates. The dotted red line denotes the model performance for 20 species, which was the number of species analyzed in the main manuscript and shown in Figure 4 and Supplemental Figures 4 and 5. (B) Scatter plot demonstrating the actual value of fecal calprotectin (y-axis) plotted against the value of fecal calprotectin as predicted by the full model (x-axis). The correlation coefficient (ρ = 0.52) is indicated at the top right corner. (C) Scatter plot demonstrating the actual value of fecal calprotectin (y-axis) plotted against the abundances of *Staphylococcus aureus* (x-axis). The correlation coefficient (ρ = 0.30) is indicated in the right top right corner. (D) Bar plots demonstrating model performance in predicting the value of fecal calprotectin (as indicated by Spearman correlation on the y-axis) for the model with *S. aureus* alone, the full model, and partial correlation subtracting the effect of *S. aureus*. Note that the full model performance possesses the highest correlation coefficient in predicting fecal calprotectin as compared to the other two models, suggesting that multiple species (and not *S. aureus* alone) are required to account for changes in the abundance of fecal calprotectin.


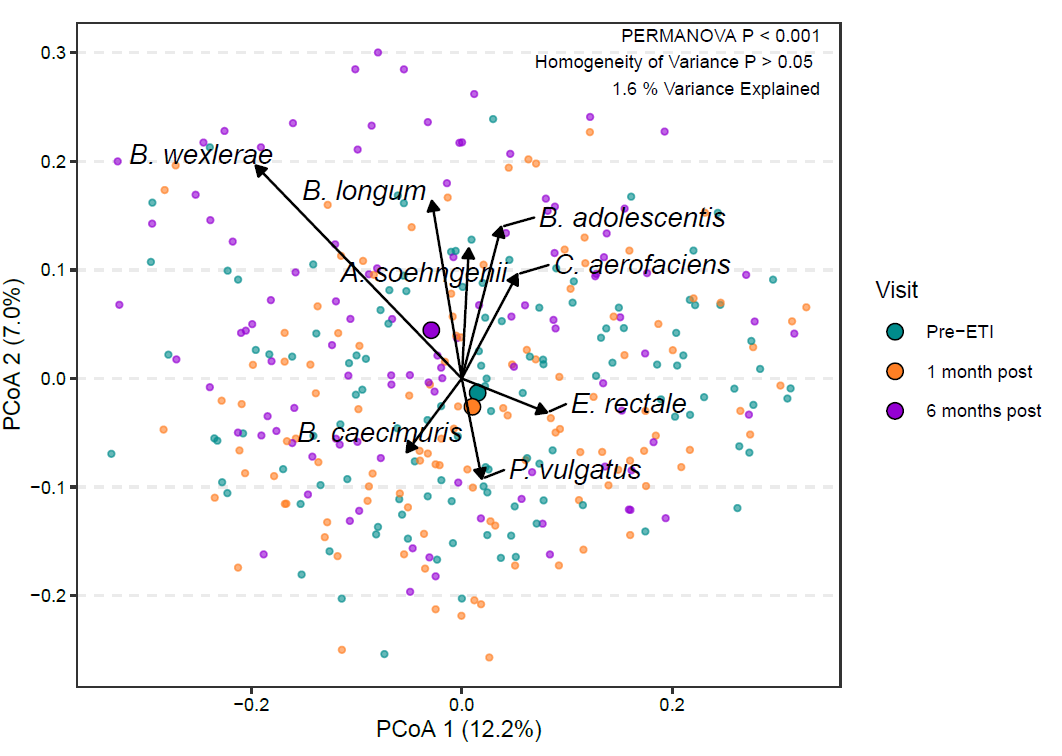


Supplemental Figure 3. Differences in the fecal microbiota are observed among participants at 6 months of ETI compared to baseline and 1 month of ETI

Principal coordinates analysis (PCoA) utilizing Bray-Curtis dissimilarity demonstrating fecal microbiota of participants pre-ETI (n=124) and at 1 (n=116) and 6 months post-ETI (n=105) as in Figure 3C. Vector arrows (black) indicating taxa contributing most to differences in microbiota between all samples. Length of the vector denotes contribution of the indicated taxon to inter-sample dissimilarities. The taxa identified include *Blautia wexlerae (B. wexlerae), Bifidobacterium longum (B. longum), Bifidobacterium adolescentis (B. adolescentis), Bacteroides caecimuris (B. caecimuris), Collinsella aerofaciens (C. aerofaciens), Eubacterium rectale (E. rectale), Phocaeicola vulgatus (P. vulgatus),* and *Anaerobutyricum soehngenii (A. soehngenii)*.


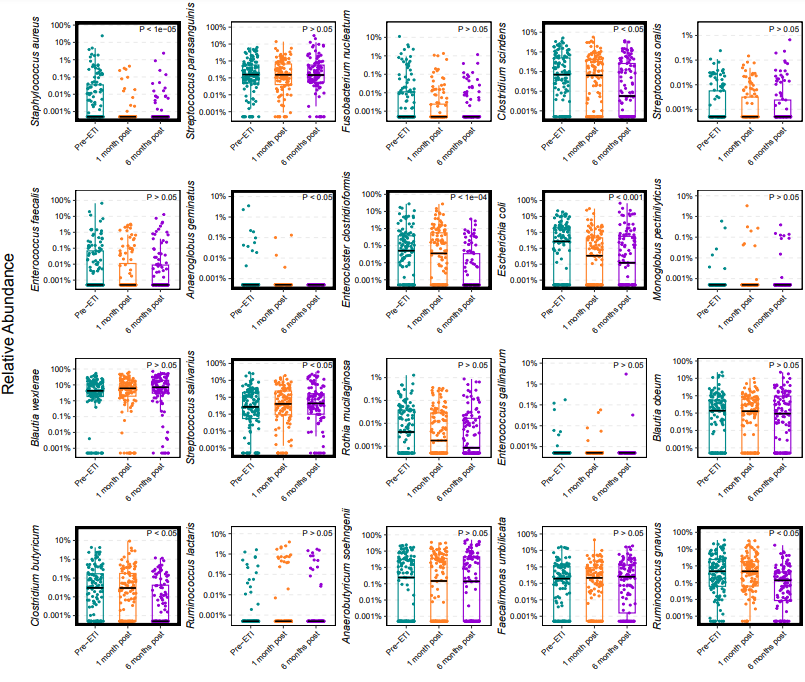


Supplemental Figure 4. Relative abundances of 8 species changed significantly after 6 months of ETI as compared to pre-ETI

Boxplots indicating the relative abundances of the top twenty species associated with fecal calprotectin (identified based on random forest importance score) before and after treatment with ETI (pre-ETI n=124, 1 month post n=116, and 6 months post n=105). The black line indicates the median and the boxplot hinges indicate the first and third quartiles. Statistical testing was performed via quantile mixed effects models. Groups of microbes linked genomically (referred to as species-level genome bins, SGBs) but without species-level taxonomic characterization were excluded. Species with significant change after ETI indicated by the black bolded boxes. Pairwise testing was performed via mixed effects model and demonstrated in Supplemental Table 4.


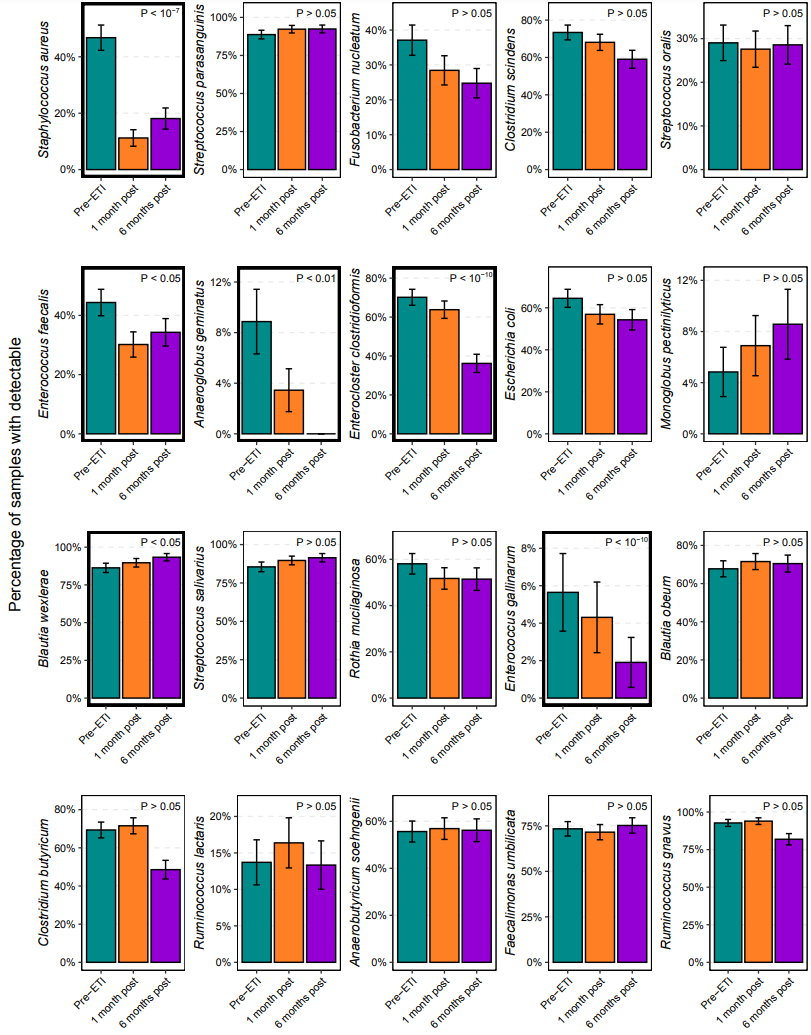


Supplemental Figure 5. The proportion of samples with detectable *Staphylococcus aureus, Enterococcus faecalis, Anaeroglobus geminatus, Enterocloster clostridioformis,* and *Enterococcus gallinarum* declined after 6 months of ETI

Bar plots demonstrating the percentage of samples with a detectable level of the indicated species (y-axis) plotted against time pre-ETI, 1 month, and 6 months post-ETI (x-axis). Species with significant change after ETI indicated by the black bolded boxes. Detectable abundance was defined as any measured abundance (> 0).


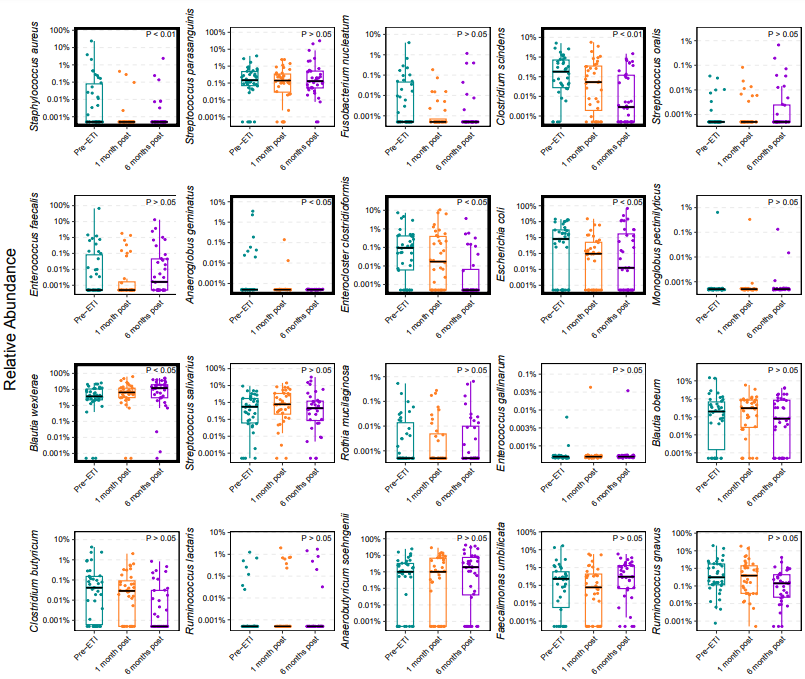


Supplemental Figure 6. In participants with initially abnormal calprotectin pre-ETI which subsequently normalized at 1-month or 6-month ETI, relative abundances of 6 species changed significantly after 6 months of ETI as compared to pre-ETI

Boxplots indicating the relative abundances of the top twenty species associated with fecal calprotectin (identified based on random forest importance score) before and after treatment with ETI (pre-ETI n=38, 1 month post n=36, and 6 months post n=36). The black line indicates the median and the boxplot hinges indicate the first and third quartiles. Statistical testing was performed via quantile mixed effects models. As in Supplemental Figure 4, SGBs without species-level taxonomic characterization were excluded. Species with significant change after ETI indicated by the black bolded boxes. Pairwise testing was performed via mixed effects model and demonstrated in Supplemental Table 5.
